# Enhanced CycleGAN Network with Adaptive Dark Channel Prior for Unpaired Single-Image Dehazing

**DOI:** 10.3390/e25060856

**Published:** 2023-05-26

**Authors:** Yijun Xu, Hanzhi Zhang, Fuliang He, Jiachi Guo, Zichen Wang

**Affiliations:** 1 Westa College, Southwest University, Chongqing 400715, China; 2 College of Electronic and Information Engineering, Southwest University, Chongqing 400715, China; 3 Chongqing Key Laboratory of Nolinear Circuits and Intelligent Information Processing, Southwest University, Chongqing 400715, China

**Keywords:** dehaze, atmospheric scattering model, cycle generative adversarial network, dark channel prior

## Abstract

Unpaired single-image dehazing has become a challenging research hotspot due to its wide application in modern transportation, remote sensing, and intelligent surveillance, among other applications. Recently, CycleGAN-based approaches have been popularly adopted in single-image dehazing as the foundations of unpaired unsupervised training. However, there are still deficiencies with these approaches, such as obvious artificial recovery traces and the distortion of image processing results. This paper proposes a novel enhanced CycleGAN network with an adaptive dark channel prior for unpaired single-image dehazing. First, a Wave-Vit semantic segmentation model is utilized to achieve the adaption of the dark channel prior (DCP) to accurately recover the transmittance and atmospheric light. Then, the scattering coefficient derived from both physical calculations and random sampling means is utilized to optimize the rehazing process. Bridged by the atmospheric scattering model, the dehazing/rehazing cycle branches are successfully combined to form an enhanced CycleGAN framework. Finally, experiments are conducted on reference/no-reference datasets. The proposed model achieved an SSIM of 94.9% and a PSNR of 26.95 on the SOTS-outdoor dataset and obtained an SSIM of 84.71% and a PSNR of 22.72 on the O-HAZE dataset. The proposed model significantly outperforms typical existing algorithms in both objective quantitative evaluation and subjective visual effect.

## 1. Introduction

With the rapid development of digital society, computer vision technology is increasingly being applied in the fields of autonomous driving, remote sensing imaging, and intelligent monitoring. However, the quality of the images acquired by photographic equipment in hazy weather is severely affected, with the target object being obscured and the image losing a lot of detailed information. Furthermore, degraded images are not conducive to subsequent high-level vision tasks. Therefore, a method for processing and clarifying hazy degraded images is highly desired.

At present, single-image dehazing has become a mainstream method for image clarification because it is cost-effective and requires no additional constraint information. Single-image dehazing methods can be classified into image enhancement, image restoration and learning-based approaches based on their mechanism.

Traditional image enhancement methods for dehazing include Retinex theory [1], histogram equalization [2], and wavelet transforms. These methods adjust the contrast and saturation of the image to achieve dehazing without considering the physical nature of haze formation. However, these global enhancements often cause the loss of some local information and perform poorly when facing hazy images in complex scenes. In recent years, many methods combining image fusion have been widely proposed. Zheng et al. [3] used gamma correction to obtain a sequence of multiple exposed images from a single hazy image, then integrated the best region of saturation using adaptive decomposition to produce a clear image. Similarly, Galdran [4] utilized a multiscale Laplacian transform to fuse artificially exposed images to achieve dehazing. Zhu et al. [5] implemented feature extraction of a single image based on the idea of image space domain transformation; these authors then used a multiscale fusion algorithm based on fast filtering and saturation curve analysis to fuse the transformed image. These image fusion-based methods have improved traditional image enhancement, but the complexity of the algorithms used in these methods is high, and there are certain limitations to the stability of the dehazing they can provide.

Image restoration [6,7,8,9] uses prior knowledge or assumptions to establish a physical model of image degradation to achieve clarity. He et al. [7] discovered the dark channel prior (DCP) and mapped it to the atmospheric scattering model [10], designing an effective haze removal method. Zhu et al. [8] revealed the connection between haze concentration, image brightness, and saturation and developed the color attenuation prior. Berman et al. [9] proposed that haze alters the original tight color clusters in RGB space and forms haze lines through atmospheric light coordinates. Wang et al. [11] estimated the transmittance based on a prior for which there exists a linear relationship between the minimum channel of a hazy image and a clear image; these authors also introduced a weakening strategy combined with a quad-tree method of subdividing additional channels to restore the atmospheric light. Physical-model-based methods have been widely adopted since they are simple and efficient. Unfortunately, this type of algorithm requires high accuracy in parameter estimation and fails in regions that do not satisfy the prior; thus, the defogging results are often accompanied by negative effects such as color distortion and halos.

Recently, learning-based methods have significantly pushed the state of the art of unpaired image dehazing. Cai et al. [12] devised DehazeNet by integrating four different traditional defogging algorithms with deep learning. Zhang et al. [13] set two sub-networks in the pyramid network to obtain the transmittance and the atmospheric light, respectively. Li et al. [14] proposed a light-weight CNN network that they combined with the atmospheric scattering model to achieve dehazing. Chen et al. [15] developed GCANet on the basis of generative adversarial networks and adopted smooth convolution instead of extended convolution to solve the problem of grid artifacts. Similarly, a series of networks [16,17,18,19] were designed to derive clear images directly from the input hazy images without considering the degradation mechanism. Compared to conventional image enhancement and prior-based haze removal models, learning-based methods have achieved great progress. However, most of these methods are trained based on paired data and rely on clear images for positive supervision. This training process of supervision leads to excessive sensitivity to samples and the poor generalization of real-world haze removal. To address this issue, various unsupervised learning methods have been proposed. Li et al. [20] presented an unsupervised, unpaired defogging algorithm based on layer disentanglement, breaking away from training on large-scale datasets. However, this algorithm often produces images with serious color distortion and poor stability during defogging. Zhao et al. [21] proposed a weakly supervised RefineDNet, which combines the dark channel prior with a learning-based method using unpaired data for adversarial learning to improve the quality of the defogged images. Li et al. [22] integrated multi-scale feature representation with an attention mechanism and designed an enhanced decoder to improve the extraction of haze information. Ding et al. [23] unified the haze removal and noise suppression tasks and introduced a region similarity fusion module to obtain the final results. The development of unsupervised defogging algorithms has significantly alleviated the overfitting problem associated with supervised methods, but the defogging results lack realism, and the network structures tend to be complex, requiring higher computational resources.

Known as a powerful tool for unpaired image processing, CycleGAN (cycle generative adversarial network) [24] is characterized by its structure, which enables images to be converted between two domains. Recently, many unpaired CycleGAN-based dehazing approaches have been widely proposed to solve the problem that paired samples are nearly unavailable in the real world. Engin et al. [25] designed a CycleDehaze system that combines a pyramid network for high-resolution images and introduces a cyclic perception loss to improve the dehazing quality. Zheng et al. [26] introduced an enhanced attention mechanism in the CycleGAN framework and applied it to the task of defogging remote sensing images. Most CycleGAN-based dehazing methods ignore the physical properties of the hazy environment; thus, the results lack realism and variability. In order to make progress on this issue, Yang et al. [27] combined CycleGAN with the atmospheric scattering model to recover the scene depth and haze density of images to improve dehazing quality and achieved better results on synthetic datasets; however, their network, with its high-complexity structure, is still limited regarding the accuracy of estimation for transmittance.

In this paper, we specifically propose a novel unpaired dehazing network termed ADCP-CycleGAN (adaptive DCP combined with CycleGAN). The network consists of two branches that implement the reconstruction of hazy and clear images, respectively. In the dehazing process, we use the scale-adaptive DCP to accurately recover the transmittance and atmospheric light and combine the variable scattering coefficient with depth to achieve a more realistic rehazing process.

The contributions of this paper can be summarized as follows:

- A novel unpaired single-image dehazing model is proposed to fuse the dark channel prior and the enhanced CycleGAN.
- An adaptive DCP is designed to rely on the Wave-ViT semantic segmentation model, and it can accurately recover the transmittance and atmospheric light.
- In the enhanced CycleGAN method, the scattering coefficient β is obtained from two different approaches in order to generate haze of various thicknesses and uneven distributions. $\beta_{1}$ is derived from the atmospheric scattering model, while $\beta_{2}$ is randomly sampled.

The article is organized as follows. Section 2 explains the preliminary knowledge of the atmospheric scattering model and the dark channel prior, as well as the basic structure of the cycle generative adversarial network. Section 3 elaborates the proposed image dehazing method based on CycleGAN with the adaptive dark channel. The experimental results, along with relevant discussions, are illustrated in Section 4. Conclusions and future work are summarized in Section 5.

## 2. Preliminaries

### 2.1. Atmospheric Scattering Model

To describe the mechanism of haze generation, McCartney et al. [10] proposed an atmospheric scattering model in 1977,

(1) $$\begin{array}{c} I(x)=J(x)t(x)+A(1-t(x)), \end{array}$$

where I(x) and J(x) indicate a hazy degraded image and a clear image, respectively. *A* is the value of the global atmospheric light, and the transmission map, t(x), can be derived from the following relationship:

(2) $$\begin{array}{c} t\left(x\right)=e^{-\beta d(x)} \end{array}$$

where β is called the scattering coefficient, which can reflect the haze density. d(x) is the depth of field.

Based on the atmospheric scattering model, a series of dehazing algorithms using prior knowledge [6,7,8,9] have been proposed. Among them, the most representative one is the dark channel prior [7] discovered by He et al.

### 2.2. Dark Channel Prior

The prior refers to certain pixels with lower intensities in at least one RGB channel as the dark channel, which can be represented as

(3) $$\begin{array}{c} J^{dark}\left(x\right)=\underset{y\in \Omega (x)}{min}\left(\underset{c\in \{r,g,b\}}{min}J^{c}\left(y\right)\right)\to 0, \end{array}$$

where $J^{c}\left(y\right)$ denotes one of the RGB channels of a clear image, and Ω(x) is a patch centered on pixel *x*. The internal transmittance of Ω(x) can be approximated as a constant provided that the patch scale is sufficiently small. Substituting this into Equation (1), a mathematical derivation gives an estimate of the transmittance:

(4) $$\begin{array}{c} \tilde{t}\left(x\right)=1-\underset{y\in \Omega (x)}{min}\left(\underset{c\in \{r,g,b\}}{min}\frac{I^{c}\left(y\right)}{A^{c}}\right) \end{array}$$

where $I^{c}\left(y\right)$ and $A^{c}$ represent the original hazy image and the atmospheric ambient light in one of the RGB components, respectively. The subtraction term in Equation (4) is actually the dark channel intensity of $\frac{I^{c}\left(y\right)}{A^{c}}$. Combined with Equation (1), a clear result can be obtained as follows:

(5) $$\begin{array}{c} J\left(x\right)=\frac{I(x)-A}{max(t\left(x\right),t_{0})}+A, \end{array}$$

in which $t_{0}$ is a tiny constant set to prevent the value of the denominator from being zero.

**Figure 1:**
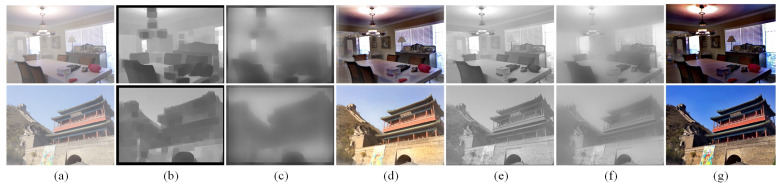
Effect of different patch sizes on dark channel prior (DCP) defogging. (**a**) Hazy input. (**b**–**d**) represent the dark channel map, transmission map, and dehazing result, respectively, based on Ω(x)=30. (**e**–**g**) are the corresponding groups, while Ω(x)=3.

The patch size of the crucial parameter Ω(x) has a decisive impact on the defogging result. As shown in Figure 1b–d, an oversized patch (Ω(x)=30) would invalidate the assumption that “the transmittance in the patch is constant”, and the patch tends to cross the edge of the depth of field, leading to the halo effect. Conversely, as shown in Figure 1e–g, if the patch scale is too small (Ω(x)=3), the intensity of the dark pixels increases; thus, the transmittance obtained from Equation (4) is less than the real value, which may result in oversaturation, distortion, and an overall darkening of the image. Therefore, a single-scale Ω(x) will cause many unexpected negative effects and reduce the image quality.

Based on this, a number of algorithms were subsequently proposed to optimize DCP performance. Chen et al. [28] proposed the concept of a “bright channel”, as opposed to the dark channel, in order to solve the problem of the misalignment of brightness in dehazing results. Zhu et al. [29] and Jackson et al. [30] introduced the energy minimization theory and Raleigh scattering theory, respectively, to remove artifacts and halos. To some extent, these approaches that introduce external theories act as a correction and complement to the original DCP, while they also undermine the advantages of DCP, i.e., its efficiency and simplicity. From the perspective of parameter adaption, Song et al. [31] compared the defogging effect at different scales in detail and adaptively adjusted the scale range of the dark channel according to the color and edge characteristics of the hazy image. Hu et al. [32] and Guo et al. [33] focused on segmenting the sky region, which does not satisfy the prior, to improve the accuracy of transmittance recovery. Inspired by previous research, we attempt to further subdivide the feature regions of the images and apply more accurate segmentation techniques to improve the quality of parameter adaptation. In Section 3.2, we will elaborate on the detailed optimization method.

### 2.3. CycleGAN

Cycle generative adversarial network was first designed by Zhu et al. [24]. It has a network structure with two generators and dual discriminators by mirror-symmetrizing the traditional GAN. Based on this special network structure, CycleGAN can convert images in the original and target domains without the supervision of paired datasets, a property that makes it widely preferred for unpaired dehazing tasks [25,26,34,35].

**Figure 2:**
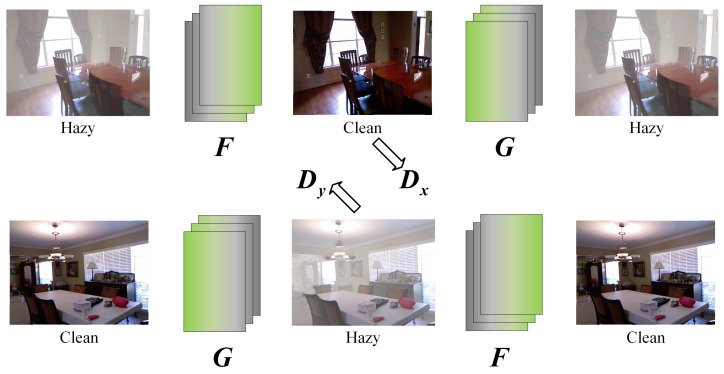
Structure of previous CycleGAN-based dehazing.

As shown in Figure 2, the previous CycleGAN-based dehazing networks contain a rehazing cycle and a dehazing cycle. In essence, most of them simply treat “hazy” and “clear” as two style domains for image transformation, with poor network interpretability and severe traces of artificial recovery. Specifically, the rehazing operation ignores real hazy environments that occur with various thicknesses and uneven distributions in the natural world, resulting in a large gap between the generated hazy images and the actual photographed hazy dataset. This means that the rehazing cycle has little significance for the enhancement of dehazing processing and can even negatively affect the quality of outputs, resulting in issues such as obvious artificial recovery traces and distortion.

In order to improve the above issues, we introduce critical physical information to realize the enhancement of the dehazing and rehazing cycle. More details will be illustrated in Section 3.1.

## 3. Proposed Method

In this section, we elaborate on an unsupervised unpaired dehazing network termed ADCP-CycleGAN. We adopt adaptive DCP to accurately recover transmittance and atmospheric light for dehazing and achieve rehazing based on the depth and scattering coefficients. The two cycle branches of hazy/clarity reconstruction are connected by the atmospheric scattering model to form enhanced CyleGAN. The algorithm and network structure are detailed as follows.

### 3.1. Network Structure

**Figure 3:**
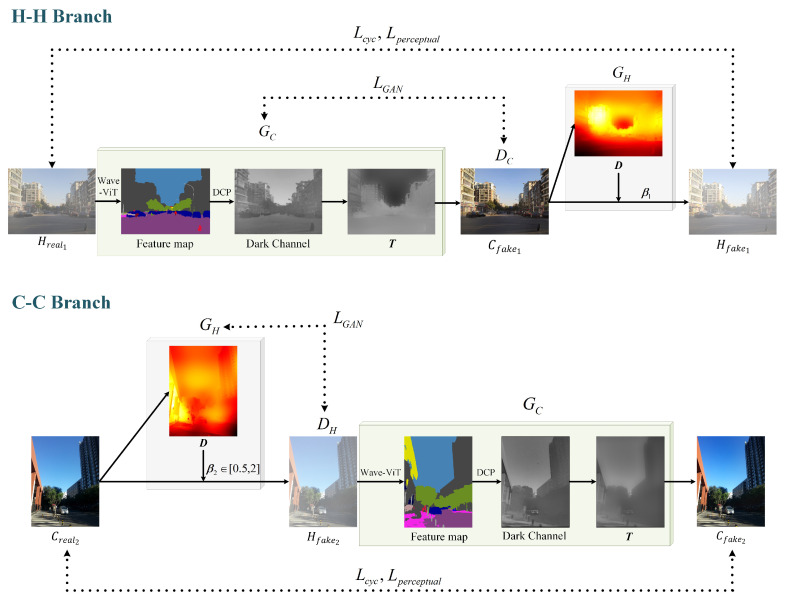
The structure of ADCP-CycleGAN.

The network consists of a hazy image reconstruction **H-H branch** and a clear image reconstruction **C-C branch**, as shown in Figure 3.

**H-H Branch**. Given a hazy image $H_{real_{1}}$, we first perform Wave-ViT segmentation of the image to obtain the regional feature map. After the DCP operation, a dark channel map is obtained to deduce the transmittance *T* and atmospheric light *A* according to Equation (4). Then, the clear image $C_{fake_{1}}$ can be acquired as follows:

(6) $$\begin{array}{c} C_{fake_{1}}=\frac{H_{real_{1}}-A}{T}+A \end{array}$$

Based on the clear image, we can restore the depth *D*, at which time the scattering coefficient $\beta_{1}$ can be recovered to reflect the density of the haze distribution. With the depth and scattering coefficients, we ultimately obtain the reconstructed hazy $H_{fake_{1}}$. In this branch, the generator $G_{C}$ is the dehazing processor, and $D_{C}$ is the discriminator that identifies whether $C_{fake_{1}}$ pertains to the clean domain.

**C-C Branch**. We initially derive the depth information from the input clear image $C_{real_{2}}$. The scattering coefficient $\beta_{2}$ is randomly sampled in the range of [0.5,2]. The corresponding hazy image $H_{fake_{2}}$ is subsequently acquired, and the same dehazing process as in the **H-H branch** is then adopted to acquire the final reconstructed clear image $C_{fake_{2}}$; that is,

(7) $$\begin{array}{c} C_{fake_{2}}=\frac{H_{fake_{2}}-A}{T}+A \end{array}$$

In this branch, the generator $G_{H}$ produces the haze, and the discriminator $D_{H}$ is used for recognizing if $H_{fake_{2}}$ belongs to the hazy domain.

### 3.2. Adaptive DCP

In Section 2.2, we discussed in detail the drawbacks of the global fixedness of Ω in DCP. In this section, we continue the idea of parameter adaption to make further improvements.

**Figure 4:**
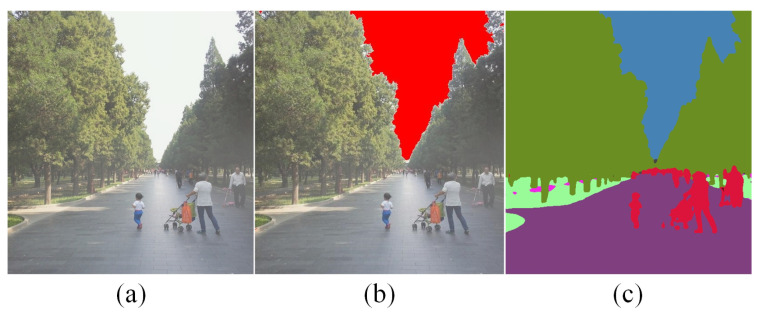
Hazy image region division. (**a**) Hazy image. (**b**) Sky region identification. (**c**) Foreground region segmentation.

To achieve a more refined segmentation of the feature regions, we here adopt the Wave-ViT model proposed by Yao et al. [36]. This model unites the wavelet transform with the Transformer network. With reversible downsampling for the lossless recovery of object texture details, it shows good performance in semantic segmentation tasks. The image division effect is shown in Figure 4.

We determine distinct patch sizes based on the essential properties of different areas in the image to achieve parameter self-adaption. The image can be divided into 3 regions: (a) The **Foreground** region, which consists of complex objects with rich colors and high saturation. An undersized patch may further aggravate the oversaturation phenomenon, whereas an oversized patch will violate the change of transmittance distribution in this region, causing an obviously distorted visual effect. Therefore, we set the patch scale of the foreground area in a normal interval that varies uniformly with the saturation. Specifically, the patch size of foreground area $\Omega_{fore}$ can be determined based on the saturation *S* and luminance *L* as follows:

(8) $$\begin{array}{c} L\left(x\right)=rI_{r}\left(x\right)+gI_{g}\left(x\right)+bI_{b}\left(x\right) \end{array}$$

(9) $$\begin{array}{c} S\left(x\right)=1-\frac{\underset{c\in \{r,g,b\}}{min}I_{c}\left(x\right)}{L(x)} \end{array}$$

(10) $$\begin{array}{c} \Omega_{fore}=max\{5,round\left(k\cdot \underset{c\in \{r,g,b\}}{min}I_{c}\left(x\right)\right\} \end{array}$$

where the max and round operators are used to set the patch scale as a positive integer. Based on previous research [7,28,31,32,33] on the defogging quality at different scales, we further conducted a validation experiment on the RESIDE dataset [37]. The results demonstrate that [5, 15] is a scale range that enables dark channel defogging to achieve optimal results, and other patch scales below or above this range experience significant negative effects, such as halos, luminance distortion, and oversaturation. Therefore, we take the value of *k* as 15 to ensure that Ω in the foreground region is adaptive in this range. As we calculate the brightness and saturation of the image in the HSI color space, the saturation value is in the range of [0, 1]. In order to change the patch scale uniformly with the saturation, we construct a linear mapping relationship between [5, 15] and [0, 1] so that pixel blocks with different levels of saturation in the foreground area can correspond to the suitable patches. (b) The **Sky** region has high brightness and low saturation. We choose a larger patch scale in the [25,30] range in this area to intensify the defogging effect. At the same time, partitioning out the region helps us find the atmospheric light values using the method in [7]. Notably, though we set the patch scale in the sky region to be much larger than in the foreground area, the sky area usually does not contain too much detail, the color saturation is more homogeneous, and the composition of the scene is simpler. At this point, the negative effects of large patches can be reduced. (c) The **Edge mutation** region. We set a smaller patch value in the range of [0, 3] in the depth of field border area to prevent halo effect and to preserve richer detail information.

### 3.3. Acquisition of Scattering Coefficient

In order to simulate the generation of real haze environments, which occur with various thicknesses and uneven distributions in the natural world, we optimize the rehazing process based on the atmospheric scattering model by combining the depth and density.

In the **H-H branch**, the scattering coefficient $\beta_{1}$ can be recovered according to Equation (2), as shown below:

(11) $$\begin{array}{c} \beta_{1}=-\frac{\ln T}{D} \end{array}$$

Based on this, the reconstructed hazy $H_{fake_{1}}$ can be described as:

(12) $$\begin{array}{c} H_{fake_{1}}=C_{fake_{1}}e^{-\beta_{1}D}+A\left(1-e^{-\beta_{1}D}\right) \end{array}$$

**Figure 5:**
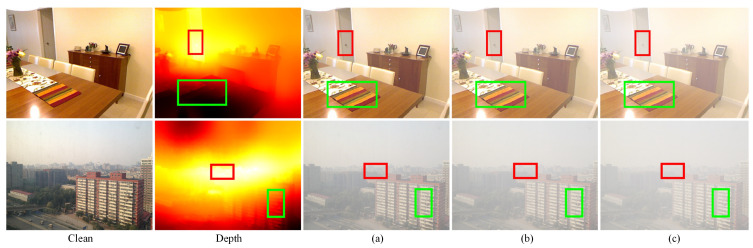
Different hazy images generated by $G_{H}$ based on diverse depth-of-field and variable scattering coefficients. (**a**) β=0.5. (**b**) β=1. (**c**) β=2.

Different from the **H-H branch**, the scattering coefficient $\beta_{2}$ in the **C-C branch** is randomly sampled in the range of [0.5,2]. By altering the scattering coefficients, the generator $G_{H}$ can produce hazy environments with arbitrary density distributions, as shown in Figure 5. Correspondingly, the hazy image $H_{fake_{2}}$ is subsequently acquired as follows:

(13) $$\begin{array}{c} H_{fake_{2}}=C_{real_{2}}e^{-\beta_{2}D}+A\left(1-e^{-\beta_{2}D}\right) \end{array}$$

It is noteworthy that, based on the atmospheric scattering model, the transmittance *T* and atmospheric light *A* derived from $G_{C}$ can be applied in $G_{H}$ to generate haze. Furthermore, these variable foggy images can also be used to augment the training of $G_{C}$. This mutually reinforcing haze removal/generation process constitutes the enhanced CycleGAN.

### 3.4. Calculation of Losses

**GAN losses** are incurred during the adversarial game between the generator and the discriminator. In our network, this occurs to ensure the quality of the dehazing and rehazing process. In the **H-H branch**, the losses of the generator $G_{C}$ and the discriminator $D_{C}$ can be expressed as follows:

(14) $$\begin{array}{c} L_{GAN}\left(G_{C}\right)=E\left[{\left(D_{C}\left(C_{fake_{1}}\right)-1\right)}^{2}\right] \end{array}$$

(15) $$\begin{array}{c} L_{GAN}\left(D_{C}\right)=E\left[{\left(D_{C}\left(C_{real_{1}}\right)-1\right)}^{2}\right]+E\left[{\left(D_{C}\left(C_{fake_{1}}\right)\right)}^{2}\right] \end{array}$$

in which $C_{fake_{1}}$ is a clear image constructed by the generator $G_{c}$, and $C_{real_{1}}$ is sampled from the clear image set Set{C}. In the **C-C Branch**, correspondingly, $H_{fake_{2}}$, which is derived from the rehazing generator $G_{H}$, and $H_{real_{2}}$, which is sampled from the hazy image Set{H}, are adopted to calculate the loss, which can be described as

(16) $$\begin{array}{c} L_{GAN}\left(G_{H}\right)=E\left[{\left(D_{H}\left(H_{fake_{2}}\right)-1\right)}^{2}\right] \end{array}$$

(17) $$\begin{array}{c} L_{GAN}\left(D_{H}\right)=E\left[{\left(D_{H}\left(H_{real_{2}}\right)-1\right)}^{2}\right]+E\left[{\left(D_{H}\left(H_{fake_{2}}\right)\right)}^{2}\right]. \end{array}$$

**Cycle-consistency losses** calculate the consistency between the original and the target domain at both ends of the loop branch. In the **H-H branch**, the input $H_{real_{1}}$ and the reconstructed hazy image $H_{fake_{1}}$ must display sufficient levels of consistency. Likewise, $C_{real_{2}}$ should agree with $C_{fake_{2}}$. Thus, the cycle-consistency losses can be written as Equation (18), where ${\left||\;|\right|}_{1}$ denotes the L1 norm.

(18) $$\begin{array}{c} L_{cyc}=E_{H_{real_{1}}∼Set\{H\}}\left|\right|H_{real_{1}}-H_{fake_{1}}{\left|\right|}_{1}+E_{C_{real_{2}}∼Set\{C\}}\left|\right|C_{real_{2}}-C_{fake_{2}}{\left|\right|}_{1} \end{array}$$

**Cycle-perceptual losses**. Although the cycle-consistency losses can be used to remove part of the noise, we also add cycle-perceptual losses to extract richer details and advanced features based on the VGG16 network to further enhance the structural similarity and ensure more realistic visual effects. The perceptual loss can be seen as Equation (19), where φ is the feature extractor and ${\left||\;|\right|}_{2}$ denotes the L2 norm.

(19) $$\begin{array}{c} L_{perceptual}=||\varphi \left(H_{real_{1}}\right)-\varphi \left(H_{fake_{1}}\right){\left|\right|}_{2}+||\varphi \left(C_{real_{2}}\right)-\varphi \left(C_{fake_{2}}\right){\left|\right|}_{2} \end{array}$$

Thus, the total loss function of ADCP-CycleGAN can be derived as:

(20) $$\begin{array}{c} L_{total}=\lambda_{1}L_{GAN}+\lambda_{2}L_{cyc}+\lambda_{3}L_{perceptual} \end{array}$$

$\lambda_{1}$, $\lambda_{2}$, and $\lambda_{3}$ are the weight-balancing factors of the three loss functions.

## 4. Experiment

### 4.1. Experimental Configuration

**Datasets**. In the experiment, four diverse datasets were adopted. (a) The RESIDE datasets [37] contain large amounts of hazy images synthesized artificially. SOTS-indoor and SOTS-outdoor contain 500 hazy/clear images indoors and outdoors, respectively, while ITS and OTS include 13,990 and 72,135 indoor and outdoor hazy and clear images, respectively. (b) The O-HAZE [38] dataset from the 2018 NTIRE Single Image Defogging Challenge contains 45 pairs of outdoor fogged/clear images with 10 pairs for testing. The images within this dataset are of high resolution and originate from real shots. (c) The BeDDE [39] dataset contains 208 real-world paired fogged/clear images of high quality captured in 23 different Chinese cities. We conducted qualitative comparison experiments on this dataset to evaluate its generalization ability and assess the subjective visual quality of real-world defogging effects. (d) In addition to the validation on the reference dataset, to compare the visual effects, we additionally introduced 30 hazy images captured in real life, as well as Fattal’s dataset [40], which contains 31 real hazy images as non-reference samples.

**Competitors Metrics**. We compared the proposed method with several state-of-art algorithms, including the most representative prior-based defogging algorithm, DCP [7]; supervised methods, including DehazeNet [12], GCANet [15], and FFANet [19]; and unsupervised methods, including ZID [20], RefineDNet [21], D4 [27], and USID [22]. For persuasive and reliable comparisons, the parameter settings were still implemented according to the content in Refs. [7,12,15,19,20,21,22,27]. We chose SSIM, PSNR [41], and LPIPS [42] as objective evaluation metrics for the dehazing performance on the reference dataset. For the test on the non-reference dataset, we focused on the evaluation of the visual effect of the dehazed image; thus, the information entropy (IE) and average gradient (AG) were employed to reflect the overall information and the local detail performance of the image, respectively. Moreover, we introduce the NIQE [43] (natural image quality evaluator) metric, which can be expressed as

(21) $$\begin{array}{c} D\left(v_{1},v_{2},\sum_{1},\sum_{2}\right)=\sqrt{{\left(v_{1}-v_{2}\right)}^{T}{\left(\frac{\sum_{1}+\sum_{2}}{2}\right)}^{-1}\left(v_{1}-v_{2}\right)}, \end{array}$$

where $v_{1}$, $v_{2}$, $\sum_{1}$, and $\sum_{2}$ represent the mean MVG value and variance matrices of the natural and distorted image, respectively. NIQE evaluates the test image by extracting features from the natural landscape, and its smaller value means the image is more compatible with human eye perception.

**Training Settings**. In the training phase, we randomly select 6000 images each from ITS and OTS, 380 images each from SOTS-indoor and SOTS-outdoor, and the training set from O-HAZE as input samples. Notably, due to the small sample size and high image resolution in the O-HAZE dataset, we cropped the 35 images into 700 copies to achieve sample expansion. All training images were rescaled to 256 × 256. We set $\lambda_{1}$, $\lambda_{2}$, and $\lambda_{3}$ as discussed in Section 3.3 to 0.2, 1, and 0.0001, respectively, to balance the weights of the three loss functions. The learning rate of the Adam optimizer was set to 0.0001, with a batch size of 2; furthermore, $\beta_{1}=0.5$, and $\beta_{2}=0.999$. We trained our model with an Nvidia GeForce RTX 2080 Ti graphics card and conducted our experiments on PyTorch.

### 4.2. Results on Reference and No-Reference Datasets

**Comparison of reference datasets**. Table 1 summarizes the average value of SSIM, PSNR, and LPIPS for every dehazing method tested on the SOTS-indoor (120 remaining images that differed from the training set, SOTS-outdoor (120 remaining images that differed from the training set), and O-HAZE datasets (10 test images cropped into 500 copies). On the SOTS-indoor test set, the supervised algorithms FFANet and GCANet demonstrate their strong capabilities and significant advantages. This is due to the fact that the supervised algorithms can sufficiently learn the image features based on paired datasets, thus performing well in simpler indoor scenes. Our proposed method achieves the best results among unsupervised algorithms. In outdoor haze removal, our algorithm performs the best among all nine algorithms on both SOTS-Outdoor and O-HAZE datasets, demonstrating that the proposed method maintains better generalization and high-quality defogging effects even in complex outdoor scenes. Meanwhile, it is worth noting that the supervised methods lose their dominant positions. To some extent, the results reflect the overfitting issues of supervised algorithms and their poor generalization abilities in handling complex scene defogging tasks.

**Figure 6:**
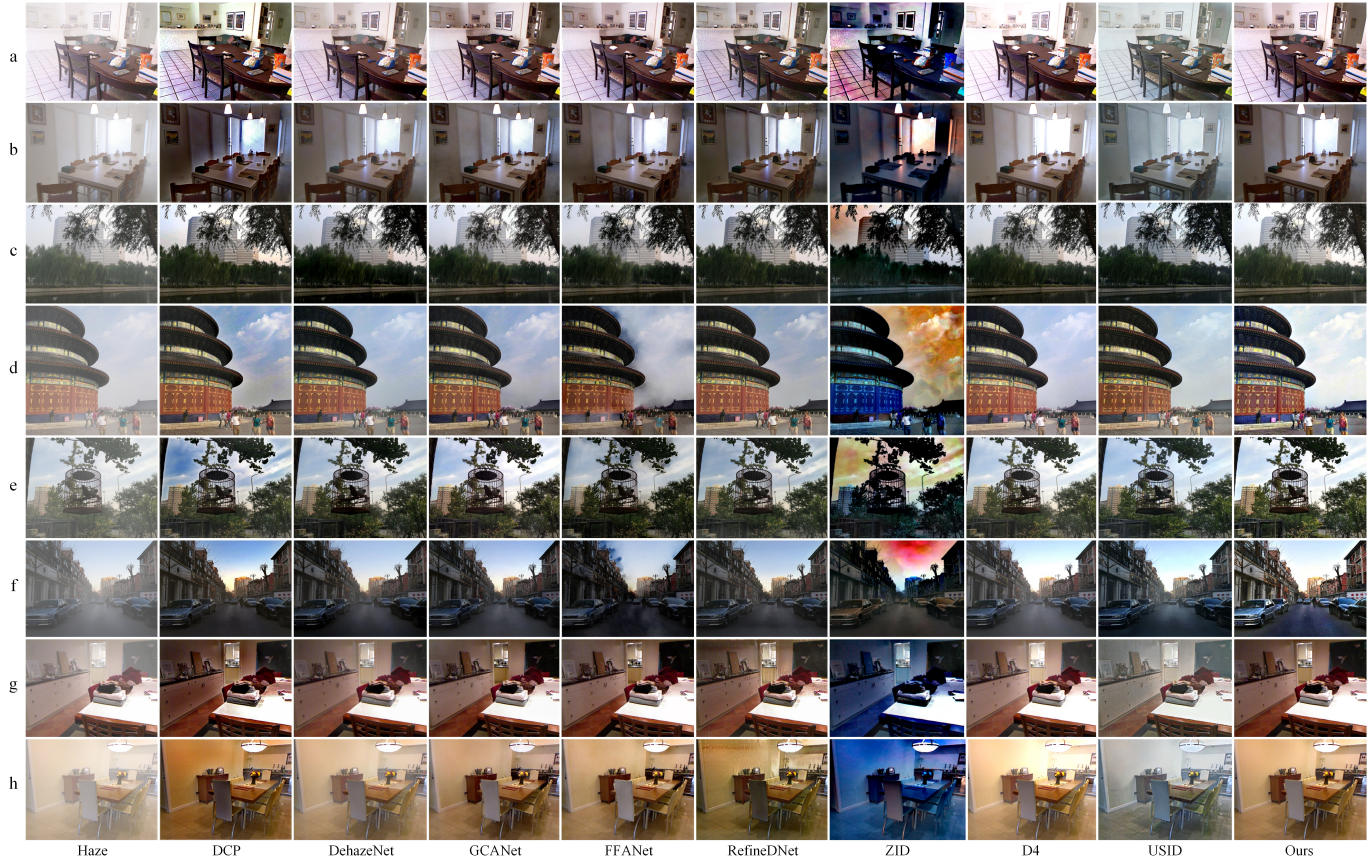
Comparative test of nine algorithms on RESIDE datasets. Rows (**a**),(**b**),(**g**),(**h**) show indoor defogging results, and rows (**c**–**f**) show outdoor defogging results. ADCP-CycleGAN dehazes well in different defogging scenarios.

Furthermore, we display visual comparisons in Figure 6. As can be observed, DCP results in an overall low brightness with obvious color distortion in the sky area. This is due to the fact that the prior is not met in the sky region. While ZID can remove haze, it suffers from significant color distortion in the fogged image. In the case of indoor defogging, RefineDNet produces some unpredictable noises in certain localized areas, such as the color block in the upper left corner of (g) and (h). The indoor defogging results of D4 suffer from serious over-brightening in the deep field due to its inaccurate estimation of atmospheric light, which is determined by taking the brightest pixel point as atmospheric light. This estimation method may lead to over-brightening of the image, especially in indoor images with artificial noise, such as light sources and mirrors. On the other hand, FFANet, GCANet, and our supervised algorithm perform better in indoor defogging, with the former two being better at preserving the details of distant indoor objects. The outdoor defogging results, as shown in Figure 6c–f, reveal the overfitting problem of FFANet, as evidenced by the noticeable color halos on the gable roof in rows d and f. The proposed method exhibits better removal of residual haze in distant parts of the image, such as the distant buildings in row f. Overall, our algorithm shows good generalization ability for various types of defogging tasks, achieving thorough defogging and satisfactory subjective visual perception.

Additionally, we compared the number of trainable parameters and the running time of our proposed ADCP-CycleGAN with other methods under the same experimental environment and summarized the results in Table 2. Of these methods, the prior-based DCP [7] does not require trainable parameters, and USID [22] outperforms the other algorithms in terms of the number of parameters and running time since it does not rely on calculating physical parameters in the atmospheric scattering model. The proposed method demonstrates acceptable network complexity and defogging efficiency, with fewer parameters and faster running speed compared to other state-of-the-art algorithms.

**Comparison on no-reference real datasets**. To verify the generalization ability of the network and the realism of the defogging results, we additionally introduced no-reference datasets. The quantitative evaluation results are reported in Table 3. Our method obtains the best scores in all three metrics, which means that the defogged images achieve acceptable results in terms of information content, detail representation, and visual effect.

**Figure 7:**
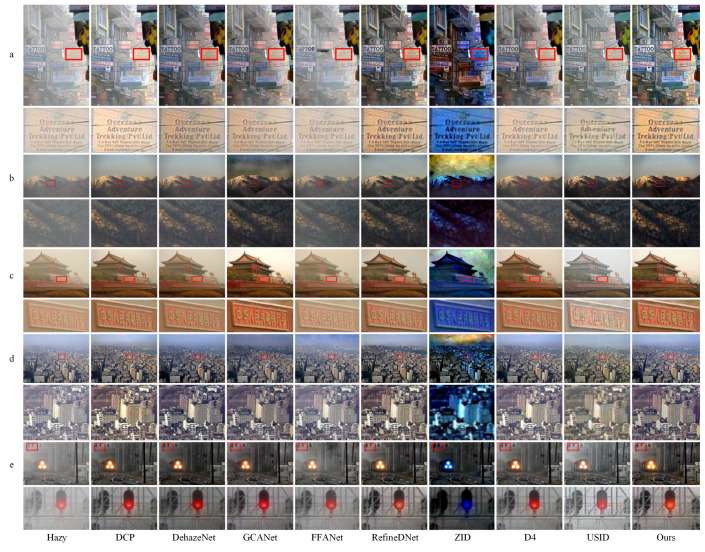
Comparative test of nine algorithms on no-reference datasets. The proposed method shows strong generalization ability and robustness in various real-world defogging tasks, with satisfactory overall picture quality and detailed information performance (**a**–**e**).

In order to demonstrate the defogging effect more clearly, we framed some local details of the image and zoomed in for comparison, as shown in Figure 7. In rows a and c, we framed the text area and zoomed in. Our method successfully preserved more edge details and restored the text information well. For the nature landscape image in row b, our method has effectively removed the residual haze, resulting in a natural color perception of the defogged image. Though GCANet also produces results with less residual haze, there are noticeable distortions in the sky region in rows b and d. FFANet defogging in real-world scenes is not desirable, as there are noticeable haze residues in the results and a large number of artifacts in the sky area of row d. In addition, the hues of USID dehazing results in rows c and d lacking naturalness and realism, resulting in poor visual effects. To summarize, our algorithm consistently shows good defogging performance on real-world no-reference datasets, providing appealing subjective and visually realistic effects.

**Figure 8:**
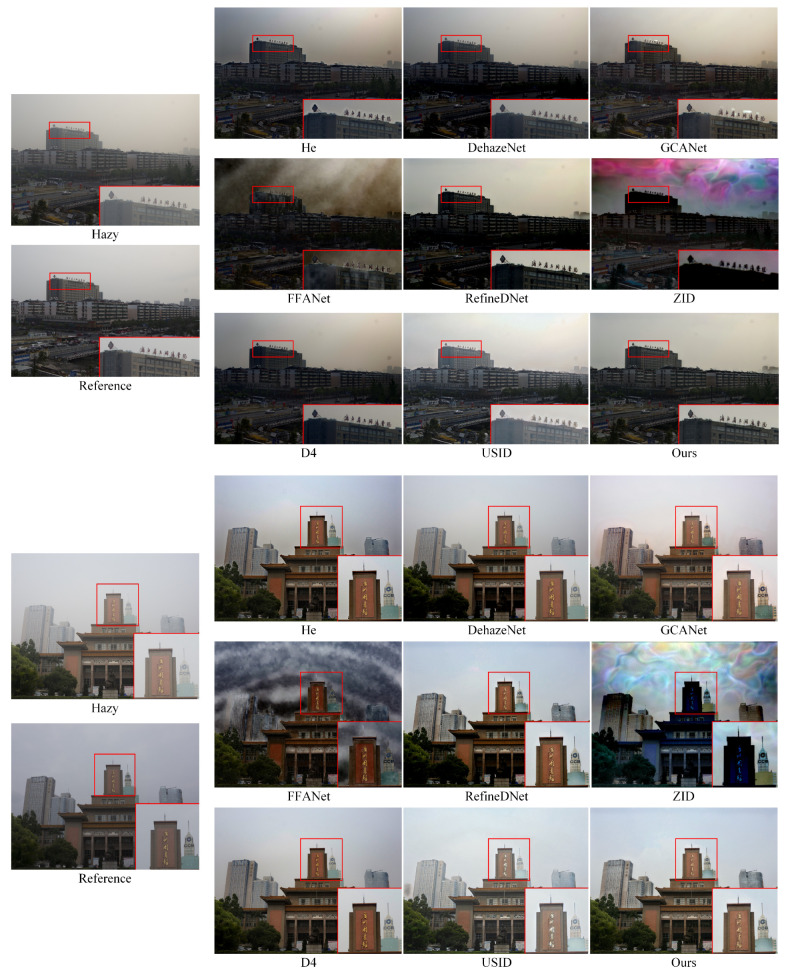
Comparative test of nine algorithms on BeDDE datasets.

**Figure 9:**
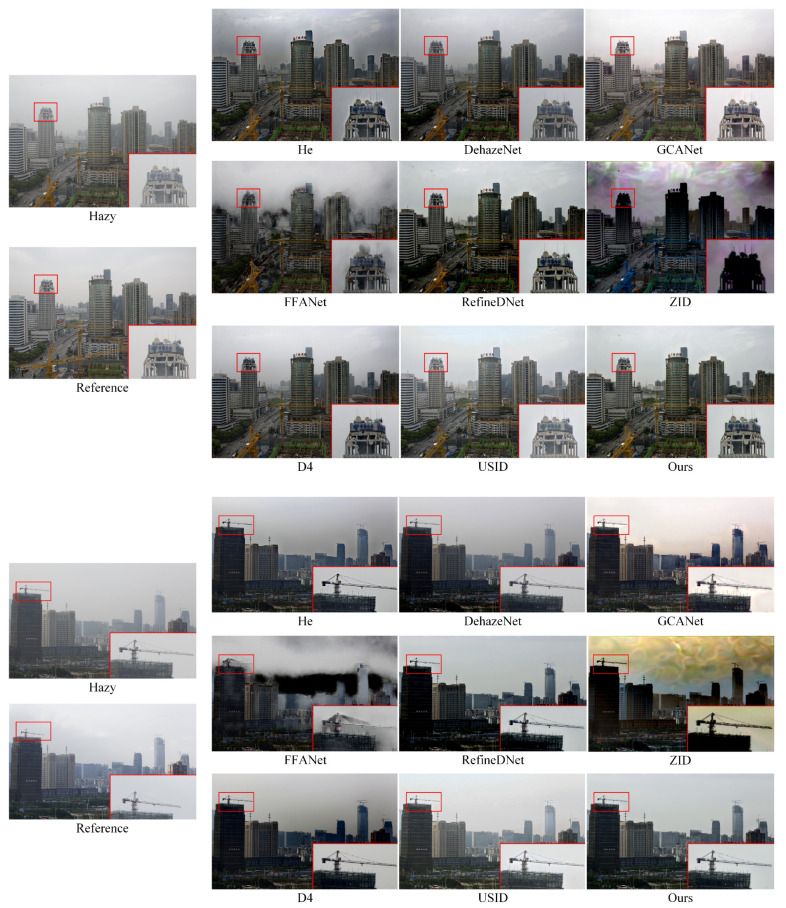
Comparative test of nine algorithms on BeDDE datasets.

In addition, we conducted abundant extended experiments on the BeDDE dataset, and the visual comparison of the defogging results is shown in Figure 8 and Figure 9. The satisfactory defogging effects further reveal the strong generalization ability and defogging stability of the proposed algorithm.

### 4.3. Ablation Study

To verify the effectiveness of the different components in ADCP-CycleGAN, we conducted an ablation study on the network. Three additional models were trained and compared with our proposed model on the SOTS dataset as follows: (a) Model A removes the Wave-ViT semantic segmentation and parameter adaptation module, and thus, the transmittance and the atmospheric light are recovered by the original DCP method; (b) the value of the scattering coefficient in Model B is set to a fixed constant; and (c) Model C deletes the cycle perceptual loss.

**Figure 10:**
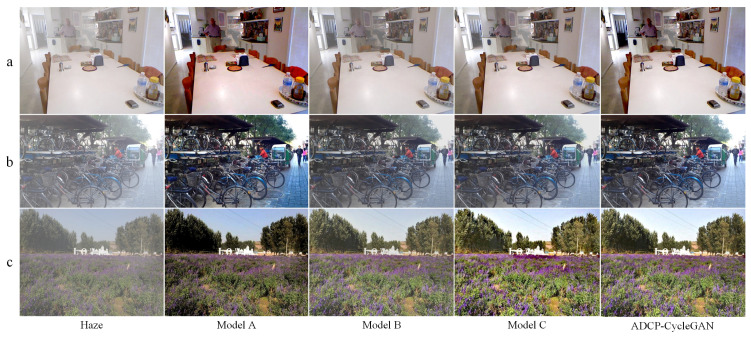
Ablation study on RESIDE datasets. The defogging results of Model A showed obvious oversaturation and color distortion. Model B showed significant haze residue. The defogging stability of Model C was reduced compared with ADCP-CycleGAN (**a**–**c**).

The dehazing results of the four models are reported in Figure 10. After removing the semantic segmentation module for the parameter adaptation of DCP, the defogging results of Model A show an obvious distortion. For the areas where the prior fails (such as the white floor tiles in row b), the distortion phenomenon appears, and the brightness of the picture in row c is also significantly darker. Model B lacks realism in the subjective visual effect of the defogging result since the scattering coefficient is set to a fixed value. Model C has a degraded performance regarding detail recovery after removing the cycle perceptual loss. The flowers in the far field in row c show an oversaturation of color, indicating that the deletion of cycle perceptual loss has an impact on defogging stability.

It is worth noting that in the quantitative analysis (as shown in Table 4), the degradation of Model B compared to ADCP-CycleGAN is different in indoor and outdoor dehazing tasks. This may due to the fact that the haze distribution in indoor scenes is more uniform compared to outdoors; thus, the scattering coefficient may have a more significant impact on the outdoor haze removal. This also confirms that the scattering coefficient is not negligible in the outdoor dehazing.

## 5. Conclusions and Future Work

In this paper, we propose ADCP-CycleGAN, a novel enhanced CycleGAN network with adaptive DCP for unpaired single-image dehazing. In the network, we achieve the parameter adaption of DCP through a Wave-ViT semantic segmentation model to recover the transmittance and atmospheric light accurately. We optimize the rehazing process by deriving the scattering coefficient from both physical calculation and random sampling means to simulate the real haze distribution. The atmospheric scattering model is applied to realize the connection between the dehazing and rehazing branch in order to build the enhanced CycleGAN. The extended experiments on both reference/no-reference datasets with diverse evaluation metrics confirm the effectiveness of our method. Specifically, our approach can generate haze that is more consistent with real-world scenarios based on depth and density. This could be particularly meaningful for tasks that require clear vision but lack unpaired datasets, such as remote sensing images, autonomous driving, and intelligent monitoring. Furthermore, we hope that our innovative combination of physical prior models with CycleGAN for dehazing can contribute to future developments in unsupervised learning for low-level vision tasks. However, there are also some aspects of our algorithm that deserve improvement. The accuracy of the depth estimation of the proposed method is affected when there is noise such as strong light and obscuration in the image. Meanwhile, due to the incorporation of a physical model in the proposed method, its inherent limitations may result in the local over-enhancement in a few defogged results. In our future work, we will also investigate the post-processing of the defogged images to further improve the image quality.

## Figures and Tables

**Table 1:** Quantitative evaluation of nine algorithms on RESIDE and O-HAZE datasets. The best score is indicated in **Bolded**.

Type	Methods	SOTS-Indoor			SOTS-Outdoor			O-HAZE		
		PSNR↑	SSIM↑	LPIPS↓	PSNR↑	SSIM↑	LPIPS↓	PSNR↑	SSIM↑	LPIPS↓
Prior	DCP [7]	16.61	0.855	0.225	19.41	0.861	0.122	12.32	0.516	0.473
Supervised	DehazeNet [12]	19.82	0.821	0.186	24.75	0.927	0.065	16.47	0.624	0.229
	GCANet [15]	30.23	0.975	0.161	24.36	0.894	0.115	18.51	0.693	0.332
	FFANet [19]	34.31	0.977	0.152	21.23	0.835	0.173	18.36	0.829	0.170
Unsupervised	RefineDNet [21]	25.06	0.929	0.199	23.58	0.914	0.047	19.27	0.853	0.152
	ZID [20]	17.26	0.801	0.244	12.19	0.614	0.396	9.82	0.437	0.528
	D4 [27]	25.40	0.934	0.207	25.75	0.936	0.035	19.90	0.844	0.147
	USID [22]	20.09	0.873	0.218	24.97	0.930	0.044	20.12	0.862	0.140
	Ours	25.98	0.941	0.157	26.95	0.949	0.031	22.72	0.871	0.136

**Table 2:** Comparison of the number of trainable parameters and average running time of different dehazing methods.

Type	Methods	Number of Parameters	Runtime (s)
Prior	DCP [7]	-	0.2930
Supervised	DehazeNet [12]	$0.008\times 10^{6}$	1.6200
	GCANet [15]	$0.660\times 10^{6}$	0.9275
	FFANet [19]	$4.964\times 10^{6}$	1.3418
Unsupervised	RefineDNet [21]	$63.378\times 10^{6}$	0.7053
	ZID [20]	$48.232\times 10^{6}$	57.3681
	D4 [27]	$11.707\times 10^{6}$	0.0579
	USID [22]	$4.022\times 10^{6}$	0.0432
	Ours	$4.275\times 10^{6}$	0.0656

**Table 3:** Quantitative evaluation of nine algorithms on no-reference datasets. The best score is indicated in **Bolded**.

Type	Methods	IE↑	AG↑	NIQE↓
Prior	DCP [7]	7.2658	7.4342	9.3858
Supervised	DehazeNet [12]	7.2945	7.0627	7.7306
	GCANet [15]	7.3098	6.4246	6.9544
	FFANet [19]	7.1056	7.1901	7.3398
Unsupervised	RefineDNet [21]	7.0903	7.9750	6.8427
	ZID [20]	7.2770	5.1849	12.4221
	D4 [27]	7.2251	7.4858	7.1425
	USID [22]	7.3560	8.0217	7.0951
	Ours	7.5238	9.8605	6.5364

**Table 4:** Quantitative evaluation of ablation study. The best score is indicated in **Bolded**.

Methods	SOTS-Indoor			SOTS-Outdoor		
	PSNR↑	SSIM↑	LPIPS↓	PSNR↑	SSIM↑	LPIPS↓
Model A	21.60	0.872	0.209	22.78	0.904	0.046
Model B	24.22	0.921	0.166	24.19	0.916	0.043
Model C	23.09	0.919	0.173	24.48	0.925	0.037
ADCP-CycleGAN	25.98	0.941	0.157	26.95	0.949	0.031

## Data Availability

Data sharing no applicable.
